# Rethinking Gain-of-Function Experiments in the Context of the COVID-19 Pandemic

**DOI:** 10.1128/mBio.01868-20

**Published:** 2020-08-07

**Authors:** Michael J. Imperiale, Arturo Casadevall

**Affiliations:** a Department of Microbiology and Immunology, University of Michigan, Ann Arbor, Michigan, USA; b Department of Molecular Microbiology and Immunology, Johns Hopkins Bloomberg School of Public Health, Baltimore, Maryland, USA

**Keywords:** biosafety, bioterrorism, policy

## Abstract

Proponents of the use of gain-of-function (GOF) experiments with pathogens with pandemic potential (PPP) have argued that such experiments are necessary because they reveal important facets of pathogenesis and can be performed safely. Opponents of GOF experiments with PPP have argued that the risks outweigh the knowledge gained. The COVID-19 pandemic demonstrates the vulnerability of human societies to a new PPP, while also validating some arguments of both camps, questioning others, and suggesting the need to rethink how we approach this class of experiments.

## EDITORIAL

At the turn of the 21st century, scientists and public health officials were concerned about a possible pandemic, with the greatest worry being the emergence of a highly pathogenic avian influenza virus. A number of human cases of infection with an avian influenza virus H5N1 strain had been reported in China, all of which appeared to be the result of direct contact between birds and people. The major reason for concern was that the mortality rate of those infected was extremely high, approximately 60%. However, those H5N1 viruses could not spread from person to person. Thus, if the H5N1 virus acquired the ability to transmit from human to human, the potential consequences could have been disastrous.

Two laboratories, the Kawaoka laboratory at the University of Wisconsin in the United States and the Fouchier laboratory at Erasmus Medical Center in the Netherlands, decided to test experimentally whether this H5N1 virus could evolve to transmit in such a way, using the well-established ferret model for transmission. Both laboratories, using different approaches, were able to isolate viruses that could spread via aerosols from one ferret to another (1, 2).

Their attempts to publish their results initiated a major debate about so-called gain-of-function (GOF) experiments with pathogens that have pandemic potential. Here, the GOF was the ability to spread from one mammalian host to another. The history of this debate has been described and analyzed extensively by us and others, and will not be repeated here (3, 4). What is relevant in 2020, however, is that one of the major concerns raised about these experiments has been that if there were an accidental release of a highly transmissible, highly pathogenic pathogen from a laboratory, it could spread very rapidly and cause significant morbidity and mortality. One analysis predicted an extremely high level of spread while another, from one of the laboratories involved in this research, reached a very different conclusion (5–7).

The arguments of that debate are relevant during the current COVID-19 pandemic because the spread of SARS-CoV-2 has uncovered a significant gap in global preparedness to handle a pathogen of this type, be it natural or laboratory derived. Most experts who have been studying and discussing preparedness agree that the source of the pathogen does not significantly change the nature of the response. Does this deficiency in handling the COVID-19 pandemic change how we as a research community should think about these GOF experiments? Our answer is yes, as follows.

We preface this discussion with the key point that we are not concerned with the notion of gain-of-function experiments writ large: many experiments in many biological systems confer an additional function on a gene or a protein or an organism. Rather, we are specifically talking about experiments involving pandemic pathogens, the experiments to which we refer with the uppercase GOF moniker.

We have argued previously that GOF experiments are sometimes the only way to address important questions about the biology of a pathogen (8). In the H5N1 situation, public health officials, including from the WHO, made the argument that it was critical to know whether this influenza virus could acquire a human-to-human transmission trait. We have therefore proposed an important criterion for proceeding with such experiments, namely, that there be a compelling medical reason to do so (4). That has not changed: one should not be performing GOF experiments simply to “see what would happen” without strong evidence that it could happen naturally. In other words, just because an experiment can be done does not mean that it should be done. We also argued that it is incumbent upon the scientific community to perform these experiments using strict biocontainment infrastructure and procedures, and we even admonished the community a few years ago after a rash of accidents with less pathogenic organisms (9).

In recent months, the argument was raised that SARS-CoV-2 may have accidentally escaped from a high-containment laboratory in Wuhan, China (10). At this time, the scientific consensus is that the virus emerged as a zoonosis whereby it jumped from an animal host, possibly bats or pangolins, to humans (11), and arguments about a laboratory origin for SARS-CoV-2 are more akin to a conspiracy theory than to a scientifically credible hypothesis. In the very unlikely event that SARS-Cov-2 had emerged by accidental escape from a lab, however, that would be a great cause for concern because the Wuhan facility was state of the art and presumably operating with a high degree of care.

Regardless of how SARS-CoV-2 found its way into humans, what is certain is that the world’s governments were caught off guard about how to respond. The ubiquitous ability of people to travel around the globe allowed the virus to spread rapidly before we knew what hit us, and even once we became aware, many countries reacted either too late or in arguably inappropriate ways, leading to many thousands of avoidable deaths.

Taking all of this into consideration, we posit three solutions moving forward. First, we reinforce our call for transparent review of all GOF experiments prior to their being commenced, to ensure that they are indeed addressing medically important questions and that GOF is the best way to obtain the answers. These discussions must be public, and decisions cannot be made behind closed doors, as it appears was the case for decisions late last year by the NIH to allow new GOF experiments on H5N1 to resume (12). A lack of openness only breeds distrust and suspicion and, if something untoward were to occur, might result in a draconian response that could have far-reaching implications for the future of all research involving pathogens.

Second, we call once again for a rededication of effort and attention to biosafety. All laboratories that carry out experiments on highly pathogenic organisms should be required to adhere to a common set of protocols and procedures, including appropriate personal protective equipment (PPE). Again, in the interest of transparency, the results of regular inspections should be made publicly available. Some may argue that following these first two recommendations might require disclosure of proprietary information, such as what is found in an application for funding from the NIH or any other agency. However, we would argue that the stakes here are high enough that some form of transparency is necessary. Most importantly, laboratories must institute strict screening measures for their workers that regularly evaluate exposure, and protocols must be in place to ensure that exposed workers do not transmit to others.

Our third solution requires a concerted effort, in the United States and worldwide, to enhance our capability to mitigate the risks posed by GOF experiments. This must be part of a broader effort to be prepared for biosecurity threats and future zoonotic threats from nature. With respect to the former, it is concerning to us that a bad actor may see the way COVID-19 has been (mis)handled as evidence that a bioweapon can be used to inflict a great degree of damage. We must have strong mitigation efforts in place, starting with the ability to detect and prevent planned attacks. Similarly, we must have a strong surveillance program that watches for zoonotic events. Such a program will require goodwill and cooperation with other countries and the WHO.

It is also essential that we develop better ways to respond to any future events. For any transmissible disease, first and foremost one would like to have a containment process in place that uses surveillance, testing, isolation, and contact tracing to prevent spread. The utility of this approach was evident and successful during the first SARS outbreak in 2003. That success has been more difficult (or, some might argue, impossible) to achieve with SARS-CoV-2 because this virus is highly contagious and can be spread prior to the appearance of symptoms. Despite this, some countries have been able to achieve an equivalent outcome by quickly locking down while the number of confirmed cases was very low (e.g., New Zealand) or extensive testing coupled with use of big data (e.g., South Korea).

Another important part of the response is the ability to test and produce therapeutics and vaccines. The global efforts to do this for COVID-19 have been extensive and impressive. Drugs that have already been approved for other indications are being tested for their ability to treat the disease: if one is efficacious, it would save a significant amount of time obtaining regulatory approval. Dozens of vaccine candidates are in development, including tried-and-true approaches such as inactivated and subunit vaccines, and new technologies such as adenovirus and RNA platforms. One of us has helped to organize an effort to use convalescent-phase serum, which contains antibodies that neutralize the virus (13).

One way to bolster these efforts would be to create a civilian equivalent of the U.S. military reserve system. The members of this reserve force could be drawn from various communities including scientists, public health experts, health care workers, ethicists, and anyone with an interest in serving society when there is a future infectious disease crisis. Like military reservists, they could dedicate time each year to train for responding as necessary and be deployed to assist federal, state, and local authorities with the numerous tasks required to contain an outbreak.

So, almost a decade after the great GOF debate of 2011 to 2012, the COVID-19 pandemic has shown that the arguments from both sides had merit. The anti-GOF camp’s central argument that these experiments were too dangerous to conduct because humanity was too vulnerable to a pandemic proved correct in the sense that the world was unprepared for COVID-19. On the other hand, the pro-GOF camp’s central argument that these experiments were necessary because we needed information on mechanisms of virulence and transmission also proved correct as humanity faced a new coronavirus with scant knowledge of how it spread and caused disease. Going forward, we need the humility to recognize that both sides had important points and find ways to obtain the information that we need to know while minimizing risks.

Humans are always going to be faced with new infectious threats. We live in an interconnected world in which deadly pathogens with the right traits have the ability to spread very rapidly. As a society, we must invest in (i) basic research to understand the biology of these microorganisms and how they interact with their hosts; (ii) applied research to develop new diagnostics, therapeutics, and preventative measures; (iii) better training for individuals working with dangerous pathogens and guidelines for monitoring potential laboratory exposures; (iv) transparent review of proposed experiments for their benefits and risks; (v) public health capacity to monitor for potential new species jumps and outbreaks; and (vi) the ability to respond more rapidly and nimbly to events when they occur. A holistic approach such as this will provide the maximum benefits to society.
